# Relationship Between Serum Osmolality and Diabetic Foot Ulcers in a US Population With Diabetes: Results From NHANES 1999–2004

**DOI:** 10.1155/ije/8820495

**Published:** 2025-12-12

**Authors:** Taotao Zhang, Peiqian Liu

**Affiliations:** ^1^ Department of Endocrinology, Fengyang People’s Hospital, Fengyang, China

**Keywords:** cross-sectional studies, diabetic foot ulcers, NHANES, serum osmolality

## Abstract

**Background:**

This study aimed to investigate the relationship between serum osmolality and diabetic foot ulcers (DFUs).

**Methods:**

We used data from the US National Health and Nutrition Examination Survey (NHANES) from 1999 to 2004. The association of each variable with DFU was explored. A multivariate logistic regression model was used to test the relationship between serum osmolality and DFU. In addition, we performed subgroup analyses to assess the reliability of the results.

**Results:**

Serum osmolality was significantly associated with DFU (*p* value < 0.05). Multivariate logistic regression analysis showed a significant association between serum osmolality and DFU (OR = 1.04, 95% CI: 1.01–1.07). Participants in the higher osmolality group (281–312 mOsm/kg) had 2.14 times higher DFU prevalence than those in the lower group (245–280 mOsm/kg) (OR = 2.14, 95% CI: 1.29–3.55). In addition, subgroup analyses and cross‐examinations confirmed that gender, age, and other variables did not significantly alter this association (all *p* values > 0.05).

**Conclusions:**

We found that higher serum osmolality was associated with increased DFU odds. Close monitoring of serum osmolality levels in populations at high risk of DFU may facilitate early identification of the occurrence of DFU.

## 1. Introduction

Diabetic foot ulcers (DFUs) are one of the severe diabetes complications, affecting more than 100 million people worldwide and, in severe cases, leading to amputation and death [1, 2]. It is a severe public health problem worldwide, which not only causes serious physical and mental harm to patients but also brings significant economic pressure to the family and society [3]. Despite advancements in DFU treatment, the rates of disability and mortality remain high [2]. Thus, early identification of risk factors for DFU is of paramount importance.

Serum osmolality is calculated from blood sodium, blood glucose, and blood urea nitrogen and is an essential indicator of the body’s hydration status [4]. It plays a vital role in maintaining fluid balance, stabilizing acid–base equilibrium, and ensuring cellular membrane stability [5]. Tingting Hu et al. found that serum osmolality has a U‐shaped relationship with the risk of all‐cause and cardiovascular mortality in patients with diabetes [6]. Jiayu Zhang et al. found that higher serum osmolality is associated with a raised risk of diabetic retinopathy (DR) in adults [7]. Yayun He et al. discovered that elevated serum osmolality raises the risk of diabetic nephropathy and mortality in patients with diabetes [8]. Crucially, no studies have found a relationship between serum osmolality and DFU. Therefore, we aim to fill this gap by investigating this relationship using the National Health and Nutrition Examination Survey (NHANES) data.

## 2. Methods

### 2.1. Data Sources

This study analyzed data from three 2‐year NHANES cycles (1999–2004) involving US patients diagnosed with diabetes. This is because data on DFU were only available in the questionnaires for these specific cycles. The NHANES program, administered by the National Center for Health Statistics (NCHS), comprises a series of surveys to assess the health and nutritional conditions of a representative segment of the noninstitutionalized population in the United States. The data gathering and research methodology employed an advanced multistage probability cohort design, with comprehensive details on the NHANES website (http://www.cdc.gov/nchs/nhanes.htm). The NHANES protocols and procedures received approval from the NCHS Research Ethics Review Board, and all participants provided written informed consent. Secondary analyses are exempt from review by additional institutional review boards. Additionally, this study followed the reporting criteria in the Strengthening Reporting of Observational Studies in Epidemiology (STROBE) guidelines.

### 2.2. Study Design and Population

**Figure 1:**
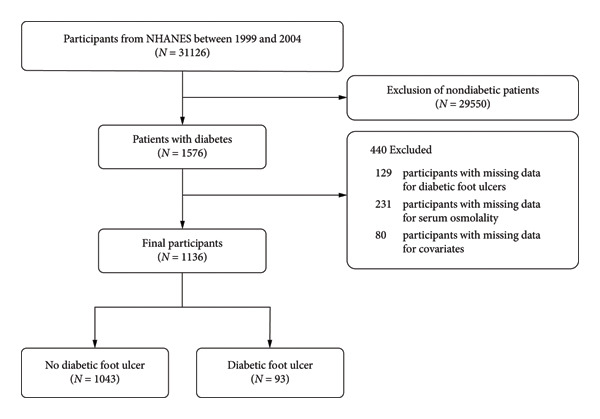
Study design and population.

We obtained data from three NHANES cycles from 1999 to 2004, with 31,126 participants completing interviews. Diabetes mellitus is defined as an individual who meets any of the following criteria: (1) fasting blood glucose ≥ 7.0 mmol/L; (2) random blood glucose or 2‐h postoral blood glucose tolerance test level ≥ 11.1 mmol/L; (3) hemoglobin A1c (HbA1c) ≥ 6.5%; (4) using insulin or hypoglycemic drugs; and (5) self‐reported diagnosis of diabetes (“the doctor told you have diabetes”). A total of 1136 participants were included in this cross‐sectional study after excluding participants without diabetes, missing DFU data, missing serum osmolality data, and missing covariates (Figure 1).

### 2.3. Serum Osmolality and DFU

Serum osmolality can be extracted directly from NHANES laboratory data. Serum osmolality was dichotomized at the median value (280 mOsm/kg). All values ≤ 280 mOsm/kg were assigned to the lower osmolality group (245–280 mOsm/kg) and values > 280 mOsm/kg to the higher group (281–312 mOsm/kg). Data on DFU were obtained from the NHANES questionnaires. Participants were classified as having a DFU if they responded “yes” to the question, “Have you ever had an ulcer or sore on your leg or foot that took more than 4 weeks to heal?”

### 2.4. Covariates

Based on the results of previous studies, we selected these covariates: age, sex, educational level, smoking status, use of insulin, DR, HbA1c, body mass index (BMI), albumin, and C‐reactive protein (CRP) [9–12]. Educational level was categorized as under 9 years of age, 9–12 years of age, and 12 years of age or older. Smoking status was categorized into three groups: never smoked (below 100 cigarettes), former smoked (smoked over 100 cigarettes but quit), and current smoker (smoked over 100 cigarettes and currently smoking). By answering the question, “Has a doctor ever told you that diabetes has affected your eyes or that you had retinopathy?” to determine if participants had DR. Participants who answered “yes” were considered to have DR. BMI was calculated as weight in kilograms divided by the square of height in meters (kg/m^2^).

### 2.5. Statistical Analysis

Sampling weights and design variables must be considered when analyzing the NHANES dataset. Ignoring these sampling parameters may result in biased estimates and inflated significance levels. Therefore, we used a complex sampling design and weights to analyze the data. The data in our study were sourced from household interviews and laboratory results in NHANES; however, the laboratory data on serum osmolality lacked specific weights. Following NHANES guidelines on survey sample weights and their proper application, we selected Mobile Examination Center (MEC) weights for our analysis [13]. The sampling weights were calculated as follows: For 1999–2002, weights were derived as 2/3 × 4‐year MEC weight, and for 2003–2004, weights were calculated as 1/3 × 2‐year MEC weight. Serum osmolality dichotomous groupings were descriptively analyzed for all participants. Qualitative data were assessed using the chi‐square test and expressed as an unweighted number (weighted percentage). Quantitative data were analyzed by *t*‐test and expressed as mean (standard deviation [SD]). Odds ratios (OR) and 95% confidence intervals (95% CIs) for the association between serum osmolality and DFU were determined using multivariable logistic regression models. Model 1 was adjusted for age, sex, and education level; Model 2 was adjusted for age, sex, education level, smoking status, use of insulin, DR, and HbA1c; and Model 3 was adjusted for age, sex, education level, smoking status, use of insulin, DR, HbA1c, BMI, albumin, and CRP. Additionally, we analyzed by sex (male vs. female), age (< 65 years vs. ≥ 65 years), HbA1c (< 6.5% vs. ≥ 6.5%), and BMI (< 30 kg/m^2^ vs. ≥ 30 kg/m^2^). All subgroup analyses were adjusted for covariates specified in Model 3, excluding the stratification variable under investigation. Interaction effects were assessed using likelihood ratio tests comparing models with and without subgroup interaction terms. Coralie Amadou et al. found higher amputation rates for DFU in men compared to women [14]. Therefore, gender was used as a stratification variable in the subgroup analysis. Weihao Chen et al. identified an elevated risk of DFU in individuals aged 65 years and older [15]. Therefore, 65 years was used as the age cutoff value in the subgroup analyses. Qian Ran et al. discovered that inadequate control of HbA1c was linked to prolonged nonhealing of DFU [16]. Therefore, a cutoff value of 6.5% was used for HbA1c in the subgroup analysis. Min‐Woong Sohn et al. found that patients with a BMI ≥ 30 kg/m^2^ have a significantly increased risk of DFU than those with a BMI of 25–29.9 kg/m^2^ [17]. Thus, a BMI cutoff value of 30 kg/m^2^ was used in the subgroup analysis. Statistical analyses were performed using Free Statistics 2.0 software (Version 2.0; Beijing, China, http://www.clinicalscientists.cn/freestatistics), and differences were considered statistically significant at a *p* value < 0.05.

## 3. Results

### 3.1. Study Design and Population

Our study utilized data from three consecutive NHANES cycles (1999–2000, 2001–2002, and 2003–2004), comprising 31,126 participants who completed the household interviews. After excluding participants without diabetes (*n* = 29,550), missing DFU data (*n* = 129), missing serum osmolality data (*n* = 231), and missing covariates (*n* = 80), a total of 1136 participants were included in this cross‐sectional study. The specific inclusion and exclusion criteria are detailed in Figure 1.

### 3.2. Characteristics of the Study Population Grouped by Serum Osmolality Dichotomy

A total of 1136 populations with diabetes (mean age 64.4 years, 51.1% male) were included. Among these, 93 cases of DFU were identified, yielding a prevalence of 8.2% (93/1136). Significant differences were observed between participants with versus without DFU in age, sex, smoking status, insulin use, DR, HbA1c, CRP, and DFU (all *p* values < 0.05) (Table 1).

**Table 1:** Characteristics of the study population grouped by serum osmolality dichotomy.

Variables	Total (*n* = 1136)	Lower (245–280) (*n* = 495)	Higher (281–312) (*n* = 641)	*p* value
Age (mean [SD])	64.4 (11.4)	62.3 (11.5)	65.9 (11.2)	< 0.001
Sex				0.005
Male	580 (51.1)	229 (46.3)	351 (54.8)	
Female	556 (48.9)	266 (53.7)	290 (45.2)	
Education level				0.413
< 9	334 (29.4)	146 (29.5)	188 (29.3)	
9–12	451 (39.7)	187 (37.8)	264 (41.2)	
> 12	351 (30.9)	162 (32.7)	189 (29.5)	
Smoking status				0.016
Never	537 (47.3)	233 (47.1)	304 (47.4)	
Current	412 (36.3)	164 (33.1)	248 (38.7)	
Former	187 (16.5)	98 (19.8)	89 (13.9)	
Use of insulin				< 0.001
No	867 (76.3)	420 (84.8)	447 (69.7)	
Yes	269 (23.7)	75 (15.2)	194 (30.3)	
DR				< 0.001
No	842 (74.1)	397 (80.2)	445 (69.4)	
Yes	294 (25.9)	98 (19.8)	196 (30.6)	
BMI (mean [SD])	30.9 (6.5)	31.1 (6.7)	30.7 (6.4)	0.342
Albumin (mean [SD])	4.2 (0.4)	4.2 (0.3)	4.2 (0.4)	0.454
HbA1c (mean [SD])	7.5 (1.9)	7.2 (1.6)	7.8 (2.0)	< 0.001
CRP (mean [SD])	0.7 (1.5)	0.8 (2.1)	0.6 (0.8)	0.039
DFU				< 0.001
No	1043 (91.8)	472 (95.4)	571 (89.1)	
Yes	93 (8.2)	23 (4.6)	70 (10.9)	

*Note:* Data for qualitative are expressed as unweighted numbers (weighted percentages), and quantitative data are expressed as means (standard deviations [SD]).

Abbreviations: BMI, body mass index; CRP, C‐reactive protein; DR, diabetic retinopathy; HbA1c, hemoglobin A1c.

### 3.3. Single‐Factor Analysis of DFU

There were significant differences in insulin use, DR, BMI, and serum osmolality between the two groups of participants (all *p* values < 0.05) (Table 2).

**Table 2:** Single‐factor analysis of diabetic foot ulcers (DFU).

Variables	Total (*n* = 1136)	No DFU (*n* = 1043)	DFU (*n* = 93)	*p* value
Age (mean [SD])	61.41 (11.70)	61.50 (11.76)	60.44 (11.10)	0.505
Sex				0.158
Male	580 (51.1)	526 (50.4)	54 (58.1)	
Female	556 (48.9)	517 (49.6)	39 (41.9)	
Education level (years)				0.552
< 9	334 (29.4)	311 (29.8)	23 (24.7)	
9–12	451 (39.7)	413 (39.6)	38 (40.9)	
> 12	351 (30.9)	319 (30.6)	32 (34.4)	
Smoking status				0.86
Never	537 (47.3)	495 (47.5)	42 (45.2)	
Current	412 (36.3)	378 (36.2)	34 (36.6)	
Former	187 (16.5)	170 (16.3)	17 (18.3)	
Use of insulin				< 0.001
No	867 (76.3)	811 (77.8)	56 (60.2)	
Yes	269 (23.7)	232 (22.2)	37 (39.8)	
DR				< 0.001
No	842 (74.1)	789 (75.6)	53 (57)	
Yes	294 (25.9)	254 (24.4)	40 (43)	
BMI (mean [SD])	31.77 (7.09)	31.63 (7.10)	33.20 (6.88)	0.0305
Albumin (mean [SD])	4.19 (0.33)	4.20 (0.33)	4.13 (0.35)	0.1366
CRP (mean [SD])	0.64 (1.20)	0.64 (1.23)	0.73 (0.89)	0.3834
HbA1c (mean [SD])	7.42 (1.82)	7.41 (1.82)	7.50 (1.85)	0.759
Serum osmolality (mean [SD])	28.13 (0.62)	28.11 (0.60)	28.36 (0.68)	0.0044

*Note:* Data for qualitative are expressed as unweighted numbers (weighted percentages), and quantitative data are expressed as means (standard deviations [SD]).

Abbreviations: BMI, body mass index; CRP, C‐reactive protein; DR, diabetic retinopathy; HbA1c, hemoglobin A1c.

### 3.4. Association Between Serum Osmolality and DFU in Multivariable Logistic Regression Models

In the fully adjusted model (Model 3: adjusted for age, sex, education level, smoking status, insulin use, DR, HbA1c, BMI, albumin, and CRP), each 1‐unit increase in serum osmolality (mOsm/kg) was associated with 4% higher odds of DFU (OR = 1.04, 95% CI: 1.01–1.07). Participants in the higher osmolality group (281–312 mOsm/kg) had 2.14 times higher DFU prevalence than those in the lower group (245–280 mOsm/kg) (OR = 2.14, 95% CI: 1.29–3.55) (Table 3).

**Table 3:** Association between serum osmolality and diabetic foot ulcers (DFU) in multivariable logistic regression models.

Variable	*N*	Model 1		Model 2		Model 3	
		OR (95% CI)	*p* value	OR (95% CI)	*p* value	OR (95% CI)	*p* value
Serum osmolality mOsm/kg	1136	1.06 (1.03–1.1)	< 0.001	1.04 (1.01–1.08)	0.026	1.04 (1.01–1.07)	0.043
Serum osmolality							
Lower (245–280)	495	1.00 (Ref)		1.00 (Ref)		1.00 (Ref)	
Higher (281–312)	641	2.54 (1.55–4.16)	< 0.001	2.16 (1.3–3.59)	0.003	2.14 (1.29–3.55)	0.003

*Note:* Lower, lower serum osmolality group (245–280 mOsm/kg); Higher, higher serum osmolality group (281–312 mOsm/kg). Model 1: adjusted for age, sex, and education level. Model 2: adjusted for age, sex, education level, smoking status, use of insulin, diabetic retinopathy, and hemoglobin A1c. Model 3: adjusted for age, sex, education level, smoking status, use of insulin, diabetic retinopathy, hemoglobin A1c, body mass index, albumin, and C‐reactive protein.

Abbreviations: 95% CI, 95% confidence intervals; OR, odds ratios; Ref, reference.

### 3.5. Subgroup Analysis

**Figure 2:**
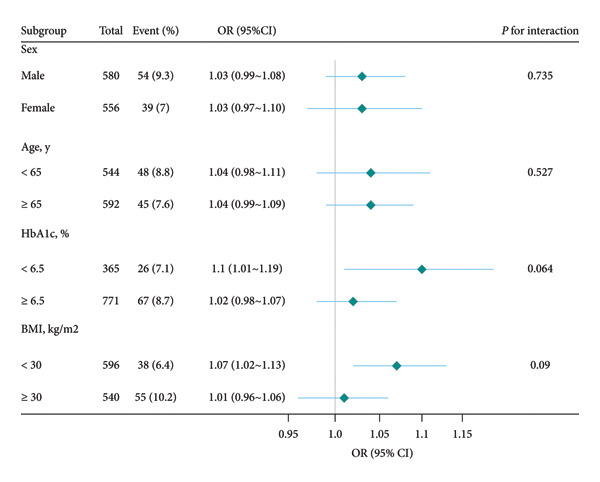
Forest plot of subgroup analyses for the association between serum osmolality and diabetic foot ulcer (DFU). *Note:* Abbreviations: OR, odds ratios; 95% CI, 95% confidence intervals; HbA1c, hemoglobin A1c; BMI, body mass index. All subgroup analyses adjusted for the full set of covariates (age, sex, education level, smoking status, use of insulin, diabetic retinopathy, hemoglobin A1c, body mass index, albumin, and C‐reactive protein), excluding the stratification variable itself in each analysis.

To explore the potential effects of sex, age, HbA1c, and BMI on the relationship between serum osmolality and DFU, stratified analyses were performed. Figure 2 shows that no significant interaction was observed in any subgroup.

## 4. Discussion

This large cross‐sectional study among US patients with diabetes demonstrates that higher serum osmolality is related to an increased risk of DFU. The results from multivariable logistic regression and subgroup analyses indicate that the relationship between serum osmolality and DFU is robust.

Serum osmolality, the osmotic pressure exerted by solutes in the serum, reflects the concentration in the blood and indicates the body’s hydration status [4]. Yinqiao Dong et al. found that, compared to the optimal hydration group, participants in the lower hydration group (HR = 1.30; 95% CI: 1.04–1.63) and the least hydrated group (HR = 1.38; 95% CI: 1.10–1.74) had a higher risk of developing diabetes [18]. Consistent with our findings, participants in the higher serum osmolality group demonstrated 2.14‐fold higher odds of developing DFUs versus those in the lower serum osmolality group (OR = 2.14; 95% CI: 1.29–3.55). Juan Carlos Parodi et al. showed that after a 6‐month hydration treatment, patients with intermittent claudication and rest pain in the lower extremities experienced improved foot skin temperature, ankle‐brachial index, claudication distance, and rest pain [19]. Yongze Zhang et al. demonstrated an association between serum sodium levels and diabetic peripheral neuropathy (DPN) [20]. DPN is among the leading causes of DFU [21]. Eric D B Goulet et al. observed that dehydration decreased lower limb muscle endurance and anaerobic capacity in elderly participants [22].

Elevated serum osmolality potentially increases DFU risk through multiple pathways. Danielle Lowry et al. found that increased serum osmolality leads to higher blood viscosity, slowing microvascular blood flow and exacerbating microcirculation dysfunction in the feet of patients with diabetes [23]. Hyperosmotic conditions promote overexpression of pro‐inflammatory cytokines such as interleukin‐6 and tumor necrosis factor‐alpha, which are mechanistically associated with DFU pathogenesis [24–26]. In hyperosmotic states, excess glucose in patients with diabetes induces the polyol pathway, producing sorbitol [27]. Accumulated intracellular sorbitol causes osmotic influx of water, resulting in cellular edema—a process implicated in DFU development [28].

There are several limitations to consider. First, DFU data were only collected through NHANES between 1999 and 2004. Therefore, we could not use more cycles of NHANES data to validate the results further. Second, although regression models and stratified analyses were performed, the potential for residual confounding from unmeasured or unknown factors cannot be entirely ruled out. Third, due to the inherent restrictions of cross‐sectional studies, the causal relationship between serum osmolality and DFU cannot be established, necessitating further prospective studies for confirmation. In addition, our DFU definition was based on the NHANES self‐reported questionnaire item (“Have you ever had an ulcer on your leg/foot taking > 4 weeks to heal?”), which has inherent limitations: It cannot distinguish (1) active from past ulcers, nor (2) DFU from other ulcer types (e.g., venous/traumatic). These limitations should be considered when interpreting our findings, and future studies applying standard DFU phenotyping (e.g., wound duration/size documentation) may better clarify this relationship.

## 5. Conclusions

In summary, we found that higher serum osmolality is associated with a raised risk of DFU. Close monitoring of serum osmolality levels in populations at high risk of DFU is essential for early identification of the occurrence of DFU.

## Ethics Statement

The US National Health and Nutrition Examination Survey (NHANES) protocol received approval from both the NHANES Institutional Review Board (IRB) and the National Center for Health Statistics (NCHS) Research Ethics Review Board. Written informed consent was obtained from all participants. As this secondary analysis utilized publicly accessible deidentified data, additional IRB approval was not required.

## Conflicts of Interest

The authors declare no conflicts of interest.

## Author Contributions

Taotao Zhang: writing–review and editing, writing–original draft, methodology, investigation, formal analysis, data curation. Peiqian Liu: writing–review and editing, validation, supervision, resources, project administration, methodology.

## Funding

This research did not receive any specific grant from funding agencies in the public, commercial, or not‐for‐profit sectors.

## Data Availability

The datasets used and analyzed during the current study are publicly available from the National Health and Nutrition Examination Survey (NHANES) repository, https://wwwn.cdc.gov/nchs/nhanes/Default.aspx.
